# Appendiceal neoplasms derived from appendiceal tip remnants following appendectomy: a report of two cases

**DOI:** 10.1186/s40792-024-01936-4

**Published:** 2024-06-13

**Authors:** Yusuke Fujii, Koya Hida, Akihiko Sugimoto, Ryohei Nishijima, Masakazu Fujimoto, Nobuaki Hoshino, Hisatsugu Maekawa, Ryosuke Okamura, Yoshiro Itatani, Kazutaka Obama

**Affiliations:** 1 Department of Surgery, Kyoto University Graduate School of Medicine, 54 Shogoin Kawahara-Cho, Sakyo-Ku, Kyoto, 606-8507 Japan; 2 Department of Diagnostic Pathology, Kyoto University Hospital, 54 Shogoin Kawahara-Cho, Sakyo-Ku, Kyoto, 606-8507 Japan; 3 Department of Diagnostic Pathology, Shiga General Hospital, 5-4-30 Moriyama, Moriyama, Shiga 524-8524 Japan

**Keywords:** Appendicitis, Residual appendix, Appendiceal neoplasm, Low-grade appendiceal mucinous neoplasm, Appendiceal cancer

## Abstract

**Background:**

Neoplasms derived from remnant appendix are rarely described, with most cases arising from the appendiceal “stump”. Here, we present two surgical cases of appendiceal neoplasms derived from appendiceal “tip” remnants.

**Case presentation:**

The first patient was a 71-year-old man who had undergone laparoscopic appendectomy for acute appendicitis 12 years prior. During appendectomy, the appendiceal root was ligated, but the appendix was not completely removed due to severe inflammation. At the most recent presentation, computed tomography (CT) was performed to examine choledocholithiasis, which incidentally revealed a cystic lesion of approximately 90 mm adjacent to the cecum. A retrospective review revealed that the cystic lesion had increased in size over time, and laparoscopic ileocecal resection was performed. Pathology revealed no continuity from the appendiceal orifice to the cyst, and a diagnosis of low-grade appendiceal mucinous neoplasm (LAMN) was made from the appendiceal tip remnant. The patient was discharged without complications. The second patient was a 65-year-old man who had undergone surgery for peritonitis due to severe appendicitis 21 years prior. During this operation, the appendix could not be clearly identified due to severe inflammation; consequently, cecal resection was performed. He was referred to our department with a chief complaint of general fatigue and loss of appetite and a cystic lesion of approximately 85 mm close to the cecum that had increased over time. CT showed irregular wall thickening, and malignancy could not be ruled out; therefore, laparoscopic ileocecal resection with D3 lymph node dissection was performed. The pathological diagnosis revealed mucinous adenocarcinoma (TXN0M0) arising from the remnant appendiceal tip. The patient is undergoing follow-up without postoperative adjuvant chemotherapy, with no evidence of pseudomyxoma peritonei or cancer recurrence for 32 months postoperatively.

**Conclusions:**

If appendicitis-associated inflammation is sufficiently severe that accurate identification of the appendix is difficult, it may remain on the apical side of the appendix, even if the root of the appendix is ligated and removed. If the appendectomy is terminated incompletely, it is necessary to check for the presence of a residual appendix postoperatively and provide appropriate follow-up.

## Background

In rare cases, the residual appendix left after appendectomy can cause inflammation or neoplastic transformations. Although residual appendicitis and tumors derived from the residual appendix are rare, they should not be completely ruled out in patients with a history of appendectomy. An inappropriate appendectomy can leave a long-segment stump, which can cause stump appendicitis [1, 2]. More rarely, there have been several reports on obstructive mucus retention in the lumen of the remnant appendix, forming appendiceal mucinous lesions [3–5]. As described in these reports, residual appendiceal tumors are most commonly derived from an inappropriately left appendiceal stump.

Inflammations or neoplastic mucinous lesions derived from appendiceal tip remnants are even rarer. It has been reported as lesions that are not continuous with the appendiceal root or cecum. And it was described that they were derived from tip remnants due to their locations or morphologies, pathological diagnoses, or initial surgical reports [6–11]. However, the rationales for the diagnoses as being derived from the tip remnants are often unclear. Moreover, there are no reports which described their changes over time since the initial appendectomy. Herein, we present two cases of appendiceal neoplasms derived from tip remnants after appendectomy, with a detailed clinical and pathological examination.

## Case presentation

### Case 1

A 71-year-old man was referred to our department because computed tomography (CT) performed for choledocholithiasis incidentally revealed a large cystic lesion approximately 90 mm in size adjacent to the cecum. The patient had undergone a laparoscopic appendectomy at our hospital 12 years prior. Soon after the appendectomy, the patient underwent examination for sleep apnea syndrome (SAS). Further, he had a history of total gastrectomy for early gastric cancer 6 years after the original appendectomy.

**Fig. 1:**
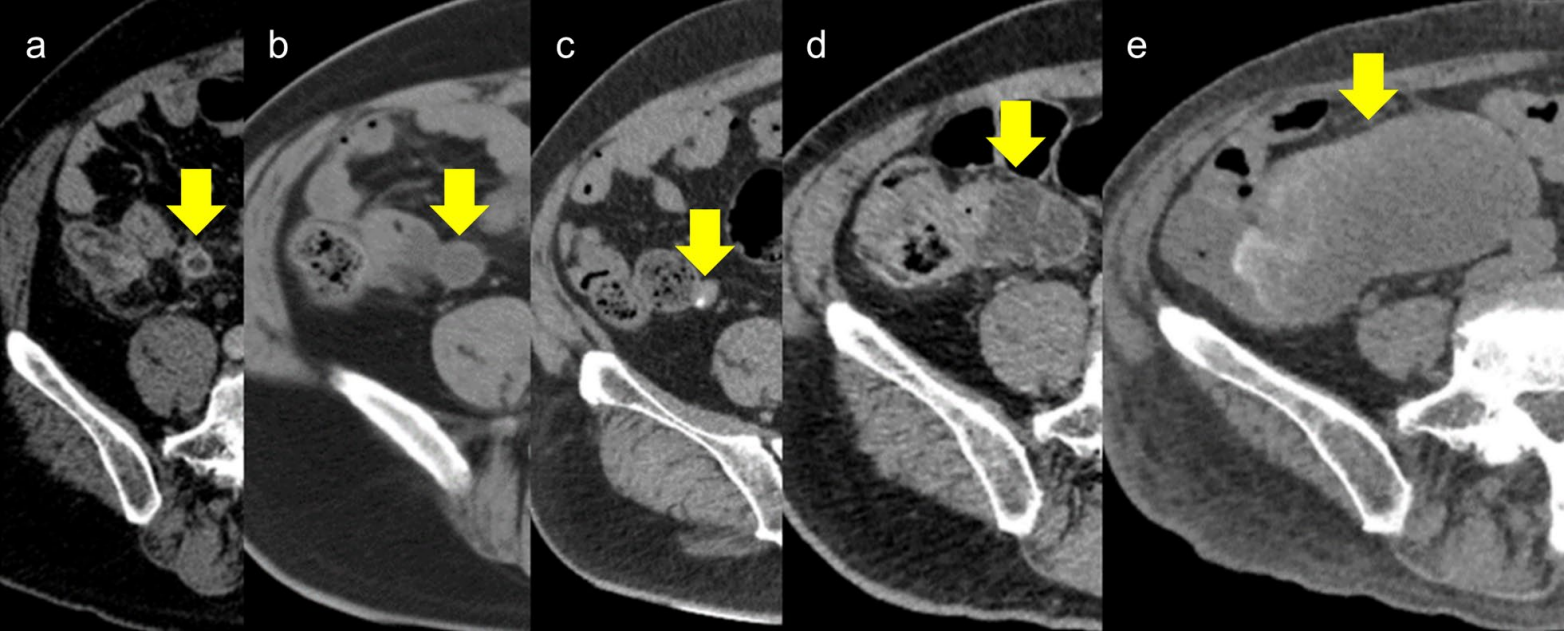
Retrospective review of the changes over time in the appendiceal tumor on CT images (Case 1). **a** The appendiceal tip could be identified just before appendectomy. **b** An appendiceal tip remnant was suspected one month after appendectomy (19 × 19 mm). **c** The tip remnant had shrunk six years after appendectomy (15 × 10 mm). **d** The tip remnant had enlarged and formed a cyst seven years after appendectomy (35 × 25 mm). **e** The cystic lesion had grown more 12 years after appendectomy (92 × 45 mm)

**Fig. 2:**
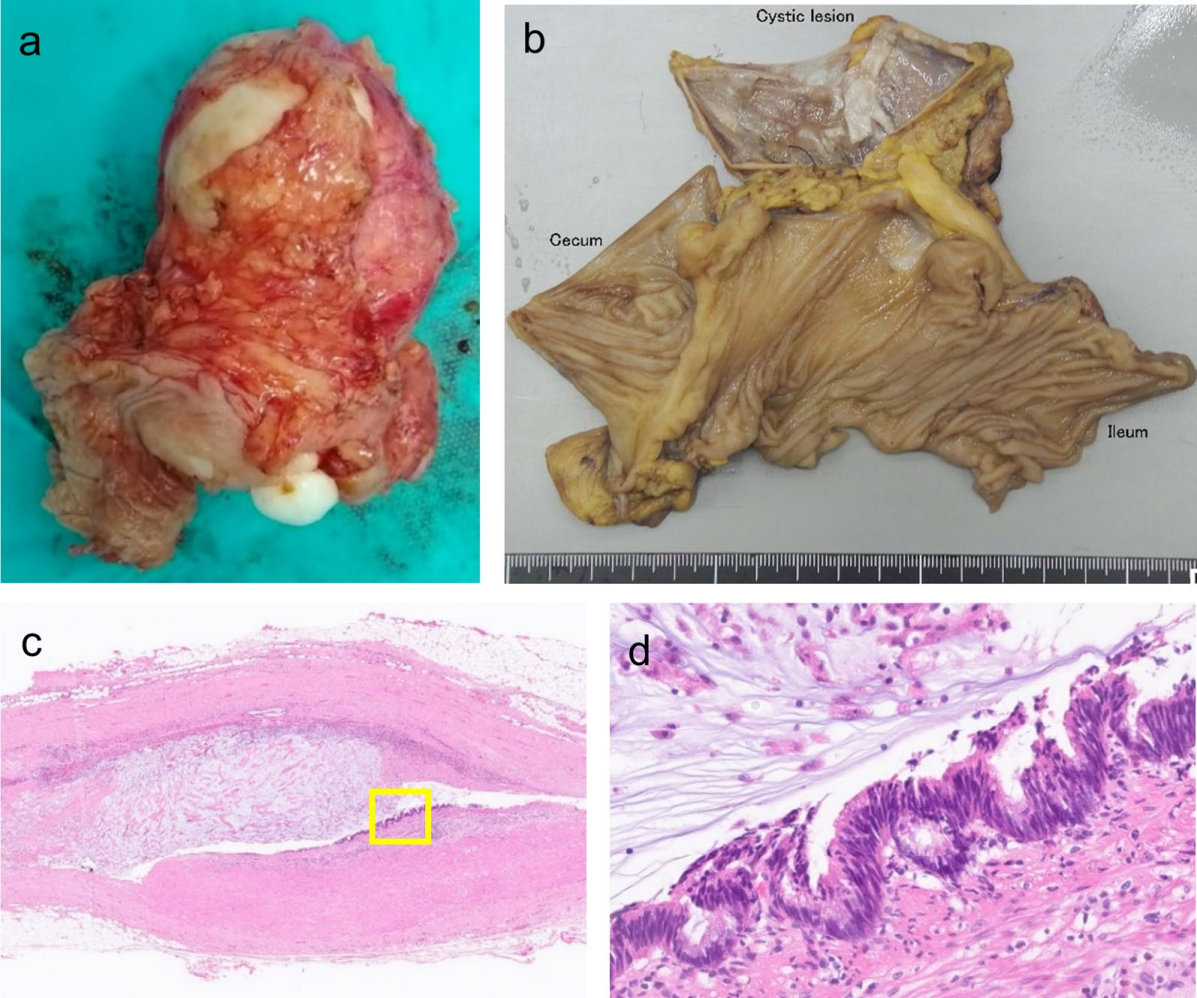
Pathological findings of case 1. **a**, **b** Macroscopic view of the specimen. The cyst was firmly adhered to the cecum, and its contents comprised white viscous mucus. There was no continuity between the cystic cavity and the cecal lumen. **c** A muscular layer was observed in the cyst wall, and mucus was present in the cavity. Most of the epithelial tissue of the cyst had fallen off, but neoplastic changes were observed in the epithelial tissue (hematoxylin–eosin stain (H.E.) × 20). **d** High columnar tumor cells with spindle-shaped enlargement of the nucleus were proliferated, and low-grade appendiceal mucinous neoplasm was observed (H.E. × 400, enlarged image of yellow square in **c**)

By reviewing the first surgical report, we found that the appendiceal root was ligated and detached; however, appendectomy was insufficiently performed because of severe inflammation. We retrospectively reviewed a computed tomography (CT) scan performed one month after appendectomy for the examination of SAS, and a remnant appendiceal tip was suspected. Six years after the appendectomy, a preoperative CT scan for gastric cancer showed that the remnant appendix has shrunk, and during the surgery for the gastric cancer, no specific findings were observed except for adhesions between the cecum and the abdominal wall. Subsequent postoperative follow-up CT revealed that the appendiceal remnant had gradually increased seven years after appendectomy and had transformed into a 92*45 mm cystic lesion (Fig. 1). We suspected an appendiceal mucinous lesion arising from the tip remnant and performed surgery. Laparoscopic observation revealed no perforation or mucus dissemination; however, the tumor and ileocecum were firmly adhered and difficult to detach. Therefore, a small incision was made in the umbilical region to perform ileocecal resection. The resected specimen showed strong adhesion between the cystic lesion and the cecum, but no continuity from the appendiceal orifice to the cyst. The cyst was found to contain white viscous mucus. Pathological findings showed that the cyst wall contained columnar epithelial and muscular tissues. Based on the medical history and pathological findings, we diagnosed the patient with a low-grade appendiceal mucinous neoplasm (LAMN) arising from an appendiceal tip remnant (Fig. 2). The patient had no postoperative complications and was discharged 7 days postoperatively.

### Case 2

**Fig. 3:**
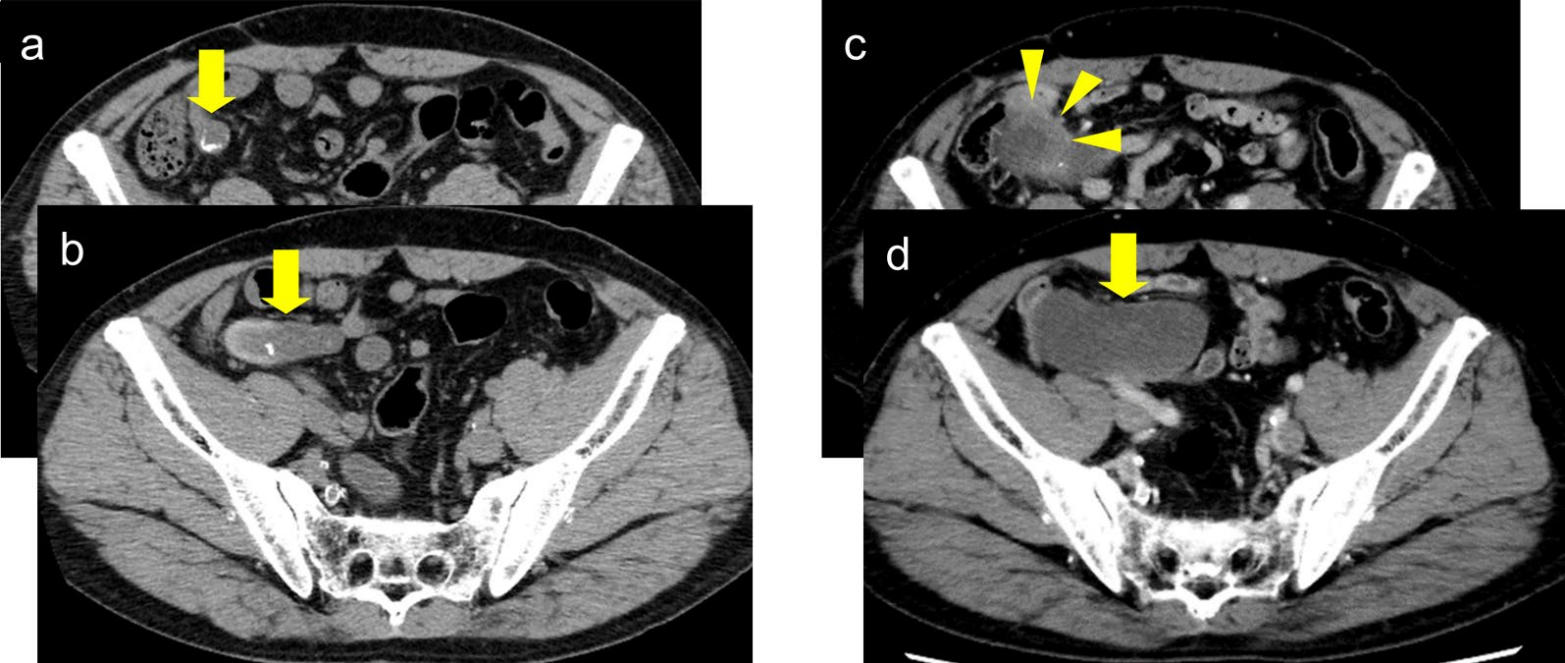
Changes of CT findings overtime in case 2. **a**, **b** A cystic lesion (55*22mm) adjacent to the ileocecum was identified 19 years after appendectomy. **c**, **d** The cystic lesion was enlarged (85*35mm) with irregular wall thickening (arrowheads) 21 years after appendectomy

A 65-year-old man visited our hospital with the chief complaint of general fatigue and loss of appetite, and was referred to our department for a tumor located in the ileocecal region. He had a surgical history of peritonitis caused by appendicitis 21 years prior. We inquired about the surgical report of the previous appendectomy, which revealed that the surgeons could not clearly recognize the entire appendix due to severe inflammation and performed open cecum resection. A contrast-enhanced CT scan revealed a cystic lesion 55 mm in size two years prior, which had grown to 85 mm in size with irregular thickening in part of the wall (Fig. 3). Tumor markers were also elevated (carcinoembryonic antigen (CEA): 83.5ng/ml, Carbohydrate antigen 19–9:260 ng/ml), and malignancy could not be ruled out. As such, surgery was performed. Laparoscopic investigation revealed that the tumor had ruptured and disseminated mucus around the cystic lesion; therefore, the patient was diagnosed as pseudomyxoma peritonei (PMP). Part of the tumor seemed to have invaded the ileum; therefore, we performed laparoscopic ileocecal resection with D3 lymph node dissection, followed by intraperitoneal lavage with 5000 mL of massive saline solution.

**Fig. 4:**
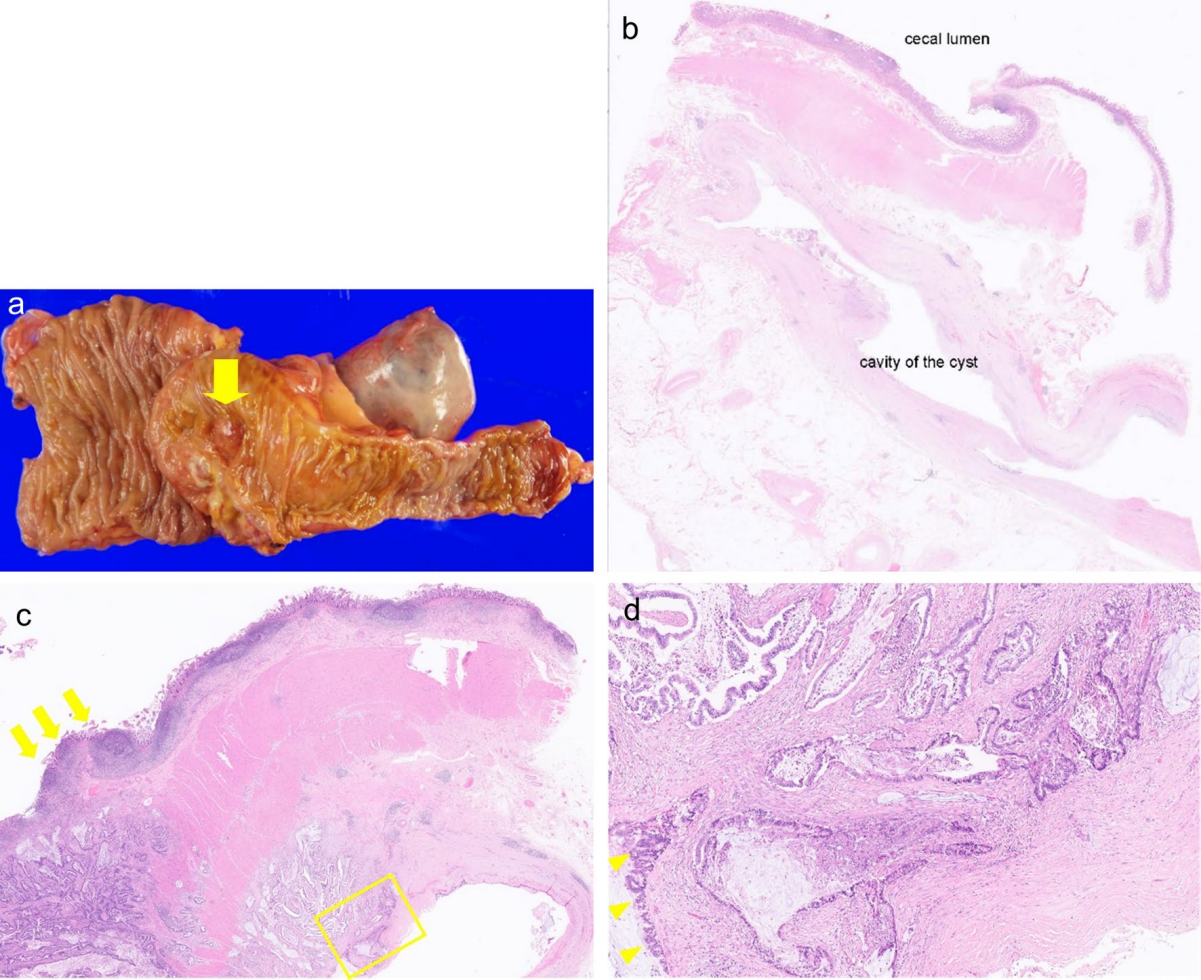
Pathological findings in case2. **a** Macroscopic view of the specimen. The cancer in the cyst wall had invaded the mucosal layer of the ileum (arrow). **b** The cystic lesion and cecum were adhered but not continued (H.E. × 5). **c** Cancer invasion of the mucosal layer of the ileum (arrows) (H.E. × 10). **d** Intraepithelial dysplasia (arrowheads) was observed in the cystic lesion, but not in the ileal mucosa; therefore, the adenocarcinoma was considered to have originated from the cystic lesion. Cancer invasion into the stromal tissue of the cystic lesion is also observed (H.E. × 100, enlarged view of the yellow square in **c**)

Examination of the resected specimen revealed that part of the tumor had invaded the ileum and extended into the mucosal layer. Immunostaining suggested the presence of desmin-positive smooth muscle and CD56- and calretinin-positive nerve cells, indicating that the cyst wall had a normal intestinal wall structure. Mucinous and well-differentiated adenocarcinomas were observed in the cyst walls (Fig. 4). No apparent cellular components were found in the mucus (acellular mucin). The cystic lesion did not continue with the stapler or the intestinal lumen; therefore, we diagnosed it as a mucinous adenocarcinoma (TXN0M0) arising from an appendiceal tip remnant. As hepatocellular carcinoma was also found postoperatively, the patient was followed-up without adjuvant chemotherapy; however, no recurrence of PMP or appendiceal cancer was observed for 32 months postoperatively.

## Discussion

The frequency of residual appendicitis after appendectomy is reported to be 0.06%–0.25% of all appendectomies [12], and only ten or more cases of appendiceal mucinous lesions derived from the residual appendix have previously been reported [11]. Histologically, appendiceal mucinous lesions are classified as either non-neoplastic or neoplastic appendiceal mucinous lesions. Non-neoplastic appendiceal mucinous lesions include simple mucoceles or retention cysts without epithelial neoplasia. Neoplastic appendiceal mucinous lesions include serrated polyps, hyperplastic polyps, low-grade appendiceal mucinous neoplasms (LAMN), high-grade appendiceal mucinous neoplasms (HAMN), and mucinous adenocarcinomas. Although there has been some controversy regarding the classification of appendiceal mucinous neoplasms, the Peritoneal Surface Oncology Group International (PSOGI) consensus or World Health Organization (WHO) 2019 classification does not recommend the use of confusing terms such as mucinous cystadenoma or mucinous cystadenocarcinoma, as previously reported [13, 14]. Indeed, the term mucinous adenocarcinomas should only be used if accompanied by infiltrative invasion, and should be classified without histologically destructive features: low-grade epithelial dysplasia as LAMN, and high-grade epithelial dysplasia as HAMN. Appendiceal mucinous lesions have poor prognosis if they become cancerous or rupture and progress to PMP [4, 15–20]. In a prior report, Tomida et al. reviewed 10 cases of mucinous lesions derived from the residual appendix that developed 10–40 years after appendectomy [11]; however, changes in tumors over time were not described in these reports. In our first case, we observed CT images taken for other diseases. It is interesting to note that the change in the size of the remnant appendix, which shrunk after the inflammation subsided, increased over time 7 years postoperatively.

Residual appendicitis or appendiceal tumors reported in previous studies are largely derived from the appendiceal stump [11, 12, 21]; those derived from appendiceal tip remnants are very rare. To the best of our knowledge, there have been nine reported cases, including the two reported here (Table 1). The incidence of residual tip appendicitis ranged from three months to ten years after the initial surgery. With regard to tumor development from the residual appendiceal tip, none developed cancer, and the time to occurrence ranged from 9 to 24 years after the initial surgery. Regarding the reasons for determining that the appendiceal tip remnant was primary, one report described it as having a separate mesentery [6]; however, few reports have described it in detail [7–11].

**Table 1:** Summary of patients who had inflammations or mucinous neoplasms derived from appendiceal tip remnants

Author	Year	Age	Sex	Symptom	Diagnosis	Interval time (years)	Diameter (cm)	Operation
Johnson et al. [6]	2006	67	Male	RLQ pain	Mucinous cystadenoma	15	7	Tumor excision
O'Leary et al. [7]	2010	43	Male	RUQ pain	Residual tip appendicitis	10	2.5	Tumor excision
Parthsarathi et al. [8]	2017	13	Male	Lower abdominal pain, vomiting, fever	Residual tip appendicitis	3 months	4	Tumor excision
Boardman et al. [9]	2019	50	Male	RLQ pain	Residual tip appendicitis	1	2	Tumor excision
Djelil et al. [10]	2020	41	Female	Abdominal pain	Mucocele, pseudomyxoma peritonei	24	15	Unknown
Djelil et al. [10]	2020	59	Male	Abdominal pain	Pseudomyxoma peritonei	9	N/A	Unknown
Tomida et al. [11]	2020	48	Male	RLQ pain	Mucus-filled lesion	23	12	Ileocecal resection
Our case	2024	71	Male	None	Low-grade appendiceal mucinous neoplasm	12	9	Ileocecal resection
Our case	2024	65	Male	General fatigue, loss of appetite	Mucinous adenocarcinoma	21	8.5	Ileocecal resection

RLQ: right lower quadrant, RUQ: right upper quadrant, N/A: not available

There is no disagreement regarding the diagnosis of a tumor derived from the remnant appendix if it is histologically or macroscopically confirmed that the tumor develops with continuity from the cecum or root of the appendix. However, if there is no continuity, whether the tumor is derived from the remnant appendix may be questioned. If there was no continuity with the root of the appendix, three differential diagnoses can be considered. The first is when the distal side of the appendix is left and the tumor develops. Second, the tumor develops due to implantation of the appendiceal mucosa or dissemination of cancer cells during the initial appendectomy [22, 23]. The third type occurs when organs other than the appendix (ovaries, duplicated intestinal cysts, ileal diverticulum, etc.) are the primary sites [24–26]. In both cases presented here, there was no continuity between the tumor and the appendiceal root or cecum. In our first case, pathological examination confirmed the presence of a gastrointestinal wall structure with a muscular layer; therefore, the possibility of mucosal implantation seemed negative. Considering the surgical report that the appendix had been incompletely resected, although the root had been removed, and the fact that the mass on the CT image 1 month after the initial surgery closely resembled the appendiceal tip on the CT image immediately before the initial appendectomy, we concluded that it was extremely likely that the appendiceal tip was left behind and developed into a tumor. In our second case, the immunostaining results revealed a normal gastrointestinal wall structure; therefore, mucosal implantation did not appear. As 21 years have passed since the initial surgery, it is unlikely that the cancer cells were disseminated at the time of appendectomy. There has been no description suggesting the presence of an enteric duplication around the ileocecum in the initial surgical report. Furthermore, the non-neoplastic part of the epithelium covering the cystic cavity was not composed of small intestinal mucosa, which contains relatively few goblet cells, but of colonic mucosa, which consist of goblet cells almost entirely. This suggests that the cystic lesion was derived from the colon (including the appendix) rather than the ileum. Actually, true diverticulum with a muscular layer is not common in the terminal ileum, which is another underlying reason why we did not conceive the lesion was derived from ileal diverticulum. Based on these points, we concluded that the patient had undergone cecal resection as the initial surgery; however, the tip of the appendix was likely left behind.

Here, the question arises regarding the blood flow that allows the remnant appendix detached from the root to become a tumor. However, it has been suggested that the colonic mucosa is highly viable and can survive and continue to produce mucus, even when cut using a stapler and blood flow is lost [27]. In another case, Aida et al. reported that mucosal implantation grew on the abdominal wall and became a tumor [23]. In addition, Johnson et al. reported a case in which the appendiceal tip was left with a clear mesentery [6], and it is possible that the mesoappendix was also left in our case. Furthermore, it was possible that the remnant tip appendix received blood supply by adhesion to nearby organs, as reported by Parthsarathi et al. and Boardman et al. [8, 9].

In terms of treatment, regardless of whether the tumor is derived from the tip or the stump, the indications for surgery for appendiceal mucinous lesions remain the same. The tumor in our first case was an LAMN which developed 12 years after the initial surgery, and the tumor in our second case was a cancer that had ruptured 21 years after the initial surgery. Surgical removal of cystic lesions is essential before they rupture or develop into cancer. In addition, gentle intraoperative manipulation is required because rupture of mucinous lesions can lead to PMP. Recently, several cases of laparoscopic resection have been reported [8, 28]. As demonstrated in this case, the strategy of performing laparoscopic adhesiolysis and ileocecal mobilization, followed by open observation and resection of the tumor, is considered effective. Lymph node metastasis is rare [29] in cases of LAMN, and lymph node dissection is therefore generally considered unnecessary. In cases of malignancy, lymph node dissection is considered, but it has also been reported that lymph node metastasis is rarely observed in patients with well-differentiated mucinous adenocarcinoma and a Tis or T1 depth of invasion (Union for International Cancer Control 8th edition) [30]. However, it is challenging to differentiate between benign and malignant lesions using preoperative CT examinations [31], and CEA levels are believed to increase in approximately 30% of benign lesions [32, 33]. Therefore, it is not always easy to distinguish the two preoperatively.

In cases of rupture or mucinous dissemination, whether they are diagnosed as LAMN or mucinous adenocarcinoma, cytoreductive surgery plus hyperthermic intraperitoneal chemotherapy (CRS + HIPEC) should be considered [31, 32]. However, only a few facilities in Japan perform this procedure. In non-specialized facilities, the cytology of mucinous components, thorough irrigation of the abdominal cavity, and surgical incision with lavage are essential to minimize the implantation of tumor cells [33]. In our second case, extensive irrigation with a large volume of normal saline was performed intraoperatively, and there has been no recurrence for 32 months postoperatively, despite the absence of postoperative chemotherapy. PMP prognosis is believed to be associated with the presence of cellular components within the mucin and extent of mucinous seeding [31]. In our case, no distinct cellular components were detected within the mucin, and mucinous dissemination was limited to the region around the appendix, suggesting the possibility of a favorable outcome without recurrence. Continued follow-up of tumor markers and CT examinations are necessary in the future.

Fundamentally, when treating such patients, it is crucial to ensure that there is no residual disease during the initial surgery. Therefore, for cases of appendicitis complicated by periappendiceal abscesses, strategies such as interval appendectomy after initial treatments including intravenous antibiotic administration or percutaneous drainage may be effective [34, 35]. However, in cases of initial treatment failure or where inflammation remains strong, surgical intervention may be necessary. Especially in cases of severe inflammation to the extent that the whole appendix cannot be identified, clinicians should bear in mind that even after detachment at the base, remnant of the distal end may persist. If surgery is performed incompletely, appropriate follow-up is necessary. For example, by conducting a CT follow-up within a few months postoperatively, and if it confirms no residuals, we can consider concluding the follow-up. However, if residual tumors are detected, resection is basically recommended. When opting for observational management, it is recommended to remove the lesions if there are symptoms, tendencies to enlarge, or findings suggestive of neoplastic mucinous lesions. Although it is challenging to definitively distinguish between non-neoplastic and neoplastic lesions by radiologic study such as CT scan, features suggesting neoplasms such as wall calcifications, wall irregularity, diameter greater than 2cm, and absence of periappendiceal fat stranding were reported [36–38]. In any case, when observational management is chosen, follow-up may be long-term, and it is desirable to adopt an early resection strategy before rupturing or malignant transformation.

## Conclusions

Herein, we report two cases of surgery for appendiceal neoplasms derived from appendiceal tip remnants after appendectomy. These experiences suggest that in cases where precise identification of the appendix is challenging because of severe inflammation during appendectomy, postoperative confirmation of whether an appendiceal remnant exists and appropriate follow-up are deemed necessary, considering the possibility that the appendiceal tip can remain intact even after detachment of the root side.

## Data Availability

Not applicable.

## Abbreviations

CT: Computed tomography
SAS: Sleep apnea syndrome
H.E.: Hematoxylin–eosin stain
CEA: Carcinoembryonic antigen
LAMN: Low-grade appendiceal mucinous neoplasms
HAMN: High-grade appendiceal mucinous neoplasms
PMP: Pseudomyxoma peritonei
CRS: Cytoreductive surgery
HIPEC: Hyperthermic intraperitoneal chemotherapy

## References

[1] Kitamura K, Ogura T, Miyamoto R, Ishida H, Matsudaira S, Takahashi A (2023). Splenic sarcoid reaction mimicking metastases in patients after uterine cancer surgery: a report of two cases. Surg Case Rep.

[2] Kako T, Kimura M, Nomura R, Uehara S, Uematsu H, Nakaya S (2023). A case of laparoscopic sigmoidectomy using thermography for colonic blood flow assessment. Surg Case Rep.

[3] Ganti S, Sohil P (2018). Renal colic: a red herring for mucocele of the appendiceal stump. Case Rep Emerg Med.

[4] Ozgür A, Cabuk G, Nass Duce M, Cereb Tombak M, Esen K (2012). Appendiceal mucocele due to mucinous cystadenocarcinoma arising from the appendiceal stump: preoperative diagnosis based on the “onion skin sign”. Jpn J Radiol.

[5] Kim MK, Lee HY, Song IS, Lee JB, Kim GH, Yoo SM (2010). A case of a giant mucocoele of the appendiceal stump presented with a palpable mass in the right thigh: pre-operative diagnosis based on characteristic multidetector CT findings. Br J Radiol.

[6] Johnson MA, Jyotibasu D, Ravichandran P, Jeswanth S, Kannan DG, Surendran R (2006). Retention mucocele of distal viable remnant tip of appendix: an unusually rare late surgical complication following incomplete appendectomy. World J Gastroenterol.

[7] O'Leary DP, Myers E, Coyle J, Wilson I (2010). Case report of recurrent acute appendicitis in a residual tip. Cases J.

[8] Parthsarathi R, Jankar SV, Chittawadgi B, Sabnis SC, Kumar SS, Rajapandian S (2017). Laparoscopic management of symptomatic residual appendicular tip: a rare case report. J Minim Access Surg.

[9] Boardman TJ, Musisca NJ (2019). Recurrent appendicitis caused by a retained appendiceal tip: a case report. J Emerg Med.

[10] Djelil D, Dohan A, Pocard M (2020). Peritoneal pseudomyxoma after incomplete appendectomy. Pleura Perit.

[11] Tomida H, Hashimoto S, Hayashi M, Koyama M (2020). Mucus-filled lesion of a distal viable remnant tip of an appendix that developed 23 years after appendectomy. Int J Surg Case Rep.

[12] Casas MA, Dreifuss NH, Schlottmann F (2022). High-volume center analysis and systematic review of stump appendicitis: solving the pending issue. Eur J Trauma Emerg Surg.

[13] Carr NJ, Cecil TD, Mohamed F, Sobin LH, Sugarbaker PH, González-Moreno S (2016). A consensus for classification and pathologic reporting of pseudomyxoma peritonei and associated appendiceal neoplasia: the results of the peritoneal surface oncology group international (PSOGI) modified delphi process. Am J Surg Pathol.

[14] WHO Classification of Tumors Editorial Board (2019). WHO classification of tumours of the digestive system.

[15] Wrafter PF, Connelly T, Khan JS, Joyce WP (2015). Pseudomyxoma peritonei diagnosed 19 years after appendicectomy. BMJ Case Rep.

[16] Taii A, Sakagami J, Shinoda M, Taniguchi H, Tosa M, Baba T (2007). Pseudomyxoma peritonei occurring after an uneventful 23 years interval from appendectomy. Intern Med.

[17] Thompson MA, Ashton RW, Pitot HC (2004). Mucinous appendiceal adenocarcinoma presenting 5 years after appendectomy. Ann Intern Med.

[18] Mishin I, Ghidirim G, Vozian M (2012). Appendiceal mucinous cystadenocarcinoma with implantation metastasis to the incision scar and cutaneous fistula. J Gastrointest Cancer.

[19] Yeong ML, Clark SP, Stubbs RS (1989). Papillary cystadenocarcinoma of the appendiceal stump with mucocele and peritoneal metastases. Pathology.

[20] Reiter S, Rog CJ, Alassas M, Ong E (2022). Progression to pseudomyxoma peritonei in patients with low grade appendiceal mucinous neoplasms discovered at time of appendectomy. Am J Surg.

[21] Kanona H, Al Samaraee A, Nice C, Bhattacharya V (2012). Stump appendicitis: a review. Int J Surg.

[22] Yamaguchi H, Ishimaru M, Suzuki H, Yamashita H, Hatanaka K, Uekusa T (2010). Isolated abdominal wound recurrence after lymph-node dissection for appendiceal adenocarcinoma. Am J Surg.

[23] Aida N, Jingu K, Uematsu T, Kitabayashi H (2013). A case of cystadenocarcinoma arising from the incision scar of appendectomy for acute appendicitis 46 years after surgery. J Jpn Surg Assoc.

[24] Matsumoto S, Minami T, Nishioka T, Akiyama T, Maekura S (2000). Right ureteral stenosis due to endometriosis occurring in the residual appendix: report of a case. Hinyokika Kiyo.

[25] Tago T, Katsumata K, Enomoto M, Miyoshi K, Shimoda Y, Wada T (2018). Pseudomyxoma peritonei that developed by means of the perforation of the mucinous cyst adenoma originating from a duplication of the ileum. Jpn J Gastroenterol Surg.

[26] Lemahieu J, D'Hoore A, Deloose S, Sciot R, Moerman P (2013). Pseudomyxoma peritonei originating from an intestinal duplication. Case Rep Pathol.

[27] Katsumata K, Mori Y, Kawakita H, Matsuda D, Enomoto M, Aoki T (2010). A study of the incidence of implantation cyst at anastomotic sites after low anterior resection of the rectum with the double stapling technique. Langenbecks Arch Surg.

[28] Park KJ, Choi HJ, Kim SH (2015). Laparoscopic approach to mucocele of appendiceal mucinous cystadenoma: feasibility and short-term outcomes in 24 consecutive cases. Surg Endosc.

[29] González-Moreno S, Brun E, Sugarbaker PH (2005). Lymph node metastasis in epithelial malignancies of the appendix with peritoneal dissemination does not reduce survival in patients treated by cytoreductive surgery and perioperative intraperitoneal chemotherapy. Ann Surg Oncol.

[30] Takeyama H, Murata K, Takeda T, Fujii M, Kagawa Y, Kawachi H (2022). Clinical significance of lymph node dissection and lymph node metastasis in primary appendiceal tumor patients after curative resection: a retrospective multicenter cohort study. J Gastrointest Surg.

[31] Chicago Consensus Working Group (2020). Chicago consensus on peritoneal surface malignancies: management of appendiceal neoplasms. Ann Surg Oncol.

[32] Sugarbaker PH (2006). New standard of care for appendiceal epithelial neoplasms and pseudomyxoma peritonei syndrome?. Lancet Oncol.

[33] Barrios P, Losa F, Gonzalez-Moreno S, Rojo A, Gómez-Portilla A, Bretcha-Boix P (2016). Recommendations in the management of epithelial appendiceal neoplasms and peritoneal dissemination from mucinous tumours (pseudomyxoma peritonei). Clin Transl Oncol.

[34] Kristo G, Itani KMF (2019). Settling the controversy-appendectomy as the criterion for appendicitis diagnosis. JAMA Surg.

[35] Oliak D, Yamini D, Udani VM, Lewis RJ, Arnell T, Vargas H (2001). Initial nonoperative management for periappendiceal abscess. Dis Colon Rectum.

[36] Wang H, Chen YQ, Wei R, Wang QB, Song B, Wang CY (2013). Appendiceal mucocele: a diagnostic dilemma in differentiating malignant from benign lesions with CT. AJR Am J Roentgenol.

[37] Sagebiel TL, Mohamed A, Matamoros A, Taggart MW, Doamekpor F, Raghav KP (2017). Utility of appendiceal calcifications detected on computed tomography as a predictor for an underlying appendiceal epithelial neoplasm. Ann Surg Oncol.

[38] Marotta B, Chaudhry S, McNaught A, Quereshy F, Vajpeyi R, Chetty R (2019). Predicting underlying neoplasms in appendiceal mucoceles at CT: focal versus diffuse luminal dilatation. AJR Am J Roentgenol.

